# Limited cutaneous systemic sclerosis skin demonstrates distinct molecular subsets separated by a cardiovascular development gene expression signature

**DOI:** 10.1186/s13075-017-1360-7

**Published:** 2017-07-04

**Authors:** Emma C. Derrett-Smith, Viktor Martyanov, Cecilia B. Chighizola, Pia Moinzadeh, Corrado Campochiaro, Korsa Khan, Tammara A. Wood, Pier Luigi Meroni, David J. Abraham, Voon H. Ong, Robert Lafyatis, Michael L. Whitfield, Christopher P. Denton

**Affiliations:** 1 Centre for Rheumatology and Connective Tissue Diseases, University College London, London, UK; 2 University Hospitals Birmingham NHS Foundation Trust, Birmingham, UK; 3 Department of Molecular and Systems Biology, Geisel School of Medicine at Dartmouth, Hanover, NH USA; 4 Experimental Laboratory of Immunological and Rheumatologic Researches, IRCCS Istituto Auxologico Italiano, University of Milan, Milan, Italy; 5 Department of Dermatology and Venerology, University of Cologne, Cologne, Germany; 6 Division of Rheumatology and Clinical Immunology, University of Pittsburgh Medical Center, Pittsburgh, PA USA

**Keywords:** Scleroderma, Genetics, Systemic sclerosis, Vasculopathy, Microarray

## Abstract

**Background:**

Systemic sclerosis (SSc; scleroderma) is an uncommon autoimmune rheumatic disease characterised by autoimmunity, vasculopathy and fibrosis. Gene expression profiling distinguishes scleroderma from normal skin, and can detect different subsets of disease, with potential to identify prognostic biomarkers of organ involvement or response to therapy. We have performed gene expression profiling in skin samples from patients with limited cutaneous SSc (lcSSc).

**Methods:**

Total RNA was extracted from clinically uninvolved skin biopsies of 15 patients with lcSSc and 8 healthy controls (HC). Gene expression profiling was performed on a DNA oligonucleotide microarray chip. Differentially expressed genes (DEG) were identified using significance analysis of microarrays (SAM). Functional enrichment analysis of gene signatures was done via g:Profiler.

**Results:**

There were 218 DEG between lcSSc and HC samples (false discovery rate <10%): 181/218 DEG were upregulated in lcSSc samples. Hierarchical clustering of DEG suggested the presence of two separate groups of lcSSc samples: “limited 1” and “limited 2”. The limited-1 group (13 samples, 10 unique patients) showed upregulation of genes involved in cell adhesion, cardiovascular system (CVS) development, extracellular matrix and immune and inflammatory response. The CVS development signature was of particular interest as its genes showed very strong enrichment in response to wounding, response to transforming growth factor (TGF)-β and kinase cascade. Neither limited-2 samples (six samples, five unique patients) nor HC samples showed functional enrichment. There were no significant differences in demographic or clinical parameters between these two groups. These results were confirmed using a second independent cohort.

**Conclusions:**

Our study suggests the presence of molecular subsets in lcSSc based on gene expression profiling of biopsies from uninvolved skin. This may reflect important differences in pathogenesis within these patient groups. We identify differential expression of a subset of genes that relate to CVS and are enriched in fibrotic signalling. This may shed light on mechanisms of vascular disease in SSc. The enrichment in profibrotic profile suggests that dysregulated gene expression may contribute to vasculopathy and fibrosis in different disease subsets.

**Electronic supplementary material:**

The online version of this article (doi:10.1186/s13075-017-1360-7) contains supplementary material, which is available to authorized users.

## Background

Systemic sclerosis (SSc; scleroderma) represents a major clinical challenge and offers insight into fundamental processes relating to autoimmunity, fibrosis and vascular injury and pathology. It is an uncommon immune-mediated rheumatic disease with a very high clinical burden, high mortality and limited treatment options [1, 2] and provides a model for more common forms of organ-based fibrosis in the lung, liver and kidney. Recent observational cohort studies have highlighted remarkable clinical diversity in terms of pattern and extent of skin and internal organ involvement, clinical outcome and response to therapy [3–5]. Most current and emerging treatment strategies focus on intensive immunosuppression though greater understanding of the biology of disease outcomes, in particular mechanisms that determine improvement in skin and organ-based disease, may help to personalize treatment strategies more effectively [6].

The limited cutaneous subset of SSc (lcSSc) is characterised by less severe and extensive skin fibrosis but patients can develop major internal organ complications and the vascular manifestations of SSc, particularly pulmonary arterial hypertension and digital ulceration, are prominent in this subset [3]. Detailed gene expression analysis in SSc biopsies has recently been used to define molecular intrinsic subsets of the disease and to provide mechanistic insight into the pathobiology [7–14]. Interestingly, clinically uninvolved skin in the more extensive diffuse cutaneous subset of SSc has closely replicated gene expression signatures compared with biopsies from clinically involved skin [7] and a number of vasoactive genes have been identified in this way in skin from patients with the limited subset, who cluster separately from healthy controls and from patients with diffuse disease [7]. It has been shown that lesional and non-lesional lcSSc biopsies consistently cluster together and show concordance in their deregulated pathways [7, 14]. The numbers of patients with limited disease in those studies are small but the results are consistent. On this basis we sought to develop greater understanding about the gene expression abnormalities in the more prevalent lcSSc subset through detailed transcriptional analysis of skin biopsies taken from uninvolved forearm skin. We hypothesise that this may give valuable insight into the key pathogenetic processes underlying the disease and also provide potential for defining and characterising molecular subsets of lcSSc. Since the molecular subsets of SSc may also inform clinical decision-making and treatment selection, there may be additional value from extending this concept more broadly into lcSSc.

In this study, we demonstrate differential gene expression in a cohort of lcSSc patients and healthy controls and describe a distinct lcSSc subgroup not identifiable by clinical or serological assessment showing enrichment in cell adhesion, cardiovascular system (CVS) development and extracellular matrix genes. We confirm our findings in a second independent cohort of samples. The CVS development signature was significantly different between both subgroups of limited patients and a control group.

## Methods

### Inclusion criteria and study participants

Demographic information, clinical history including organ involvement, other diagnoses and extent of skin involvement, autoantibody profiles and treatments were retrieved from medical notes or obtained at the time of skin biopsy. Diagnosis of SSc was made according to the 2013 American College of Rheumatologists (ACR)/European League Against Rheumatism (EULAR) classification criteria and assignment to the limited cutaneous subset reflected the distribution and extent of skin thickening at the time of biopsy together with other typical disease characteristics [15]. Autoantibodies were measured in an accredited institutional autoimmune serology laboratory using validated commercial tests with appropriate quality control and blinded assessment of the results at the time of biopsy. In brief, antinuclear antibody (ANA) pattern was screened by indirect immunofluorescence using an HEp-2 cell substrate and further characterisation of defined extractable nuclear antigen (ENA) was by counterimmunoelectrophoresis for anti-ENA using soluble extracts from human spleen and rabbit thymus acetone powder (Pelfreez Biologicals, Rogers, AR, USA) as antigen. All patients included in this study signed informed consent for their clinical and laboratory data to be used in this clinical research project.

### Sample collection

Forearm 4-mm skin punch biopsies were performed both in patients with lcSSc and in healthy controls, and were stored for extraction in RNAlater^TM^ RNA stabilisation reagent (Ambion, Austin, TX, USA) at 4 °C overnight followed by longer-term storage at -70 °C. To avoid a potential confounding effect of different degrees of skin thickening or fibrosis, biopsy sites were selected from skin that was not clinically involved. There were no changes in pigmentation or dryness.

### Microarray processing

Tissue homogenization was performed using Qiagen TissueLyser II. RNA purification was carried out in a QIAcube with Qiagen RNeasy Fibrous Tissue Mini Kit (Qiagen, Gaithersburg, MD, USA). The Agilent 2100 Bioanalyzer (Agilent, Santa Clara, CA, USA) was used to assess the RNA integrity of samples with numbers >7. RNA concentration was measured with Thermo Scientific NanoDrop 2000 Spectrophotometer (Wilmington, DE, USA). A measure of 200 ng of total RNA was amplified and labelled with Agilent's Quick-Amp Labelling Kit. Cy3-labelled samples and Cy5-labelled Universal Human Reference RNA (Stratagene, La Jolla, CA, USA) were co-hybridized to Agilent's Human Genome (4 × 44 K) Microarrays (G4112F). Data were log2 lowess-normalised and filtered for probes with intensity ≥1.5-fold over local background in Cy3 or Cy5 channels. Data were multiplied by -1 to convert them to log2(Cy3/Cy5) ratios. Probes with >20% missing data were excluded.

### Gene expression data pre-processing

Expression data were pre-processed using GenePattern [16] modules with default settings unless stated otherwise. Missing values were imputed using the ImputeMissingValuesKNN module. Expression data were collapsed from probes to unique genes using the CollapseDataset module with the Agilent 4 × 44 K chip platform. As microarrays were processed in three separate batches, batch bias was adjusted for using GenePattern implementation of ComBat [17] by means of parametric prior method and information about lcSSc samples and controls as a covariate. Batch bias was assessed before and after ComBat using guided principal component analysis (gPCA) [18]. Expression data were adjusted by median-centering genes in Cluster 3.0 [19].

### Differential expression and functional enrichment analysis

Differentially expressed genes were identified using significance analysis of microarrays (SAM) [20]. Two-class unpaired response type was used for comparing two groups and multiclass response type was used for comparing three groups. The number of permutations was set to 500. Expression data for significantly differentially expressed genes were hierarchically clustered in Cluster 3.0 and visualized in Java TreeView [21], version 1.1.6r4. Functional enrichment analysis of gene signatures was done using g:Profiler [22] with the following settings: maximum size of functional category was set to 3500, default multiple testing correction method (g:SCS significance threshold) was used and regulatory motif and protein-protein interaction databases were excluded from the analyses.

## Results

### Subject selection and clinical characteristics

There were 23 subjects included in the discovery cohort: 15 patients with lcSSc and 8 healthy controls. The study samples were collected and analysed in two stages, first in 10 biopsies from patients with lcSSc and from 5 healthy controls that generated the initial CVS signature, and then in samples from an extended cohort with an additional 6 patients with lcSSc and 3 healthy controls. These later samples were collected independently of the first set of samples.

Demographics, clinical characteristics, organ-based disease and therapies are listed in Table 1. The demographics of the control group were broadly similar to patients in terms of age, sex and ethnicity and these are also representative of the single-centre cohort that included the sample population. All patients had established lcSSc with minimum disease duration of 2 years, and most had had the condition for more than 10 years at the time of biopsy. Patients included in the study retained the subset designation according to their early stage disease and therefore no patients who would previously have been designated as having ‘diffuse’ SSc or those who had higher skin scores in the past were included. Serological findings were again representative of a cohort of lcSSc patients but kept purposefully broad: six patients had centromere pattern staining on indirect immunofluorescence (IIF) for ANA, and four had scl-70 reactivity. Two patients had overlap Ro antibodies and two were ANA-negative; one had serological features of lupus with high double-stranded (ds) DNA antibodies and low C4. There were 6/15 patients with digital ulcers at the time of biopsy and the majority took therapy for a vascular complication, either for significant Raynaud’s phenomenon or for digital ulceration. No patients from this cohort had a diagnosis of pulmonary arterial hypertension and none had had a scleroderma renal crisis or myositis. Four had interstitial lung disease and three (more than may be expected) had an overlap diagnosis of inflammatory arthritis: these two latter diagnoses resulted in the significant use of immunosuppressive medications compared with most cohorts of lcSSc patients. The broad but characteristic range of clinical and serological features demonstrated in this cohort allowed us to identify pathogenic factors that may exist at the gene expression level but that cannot be explained by standard outpatient assessment.

**Table 1:** Demographic, clinical and serological characteristics of lcSSc patients and healthy controls

		Control subjects (*n* = 8)	All LcSSc patients (*n* = 15)
Age, median (range) years		53 (29–70)	62 (28–15)
Sex, *N* (%) female		4 (50)	12 (80)
Race, *N* (%) Caucasian		7 (87.5)	10 (100)
MRSS, median (range)			6 (3–10)
Disease duration from first non-Raynaud’s symptom, median (range) years			14 (2–40)
ANA primary pattern, *N* (%) patients	Homogenous		4 (27)
	Speckled		2 (13)
	Centromere		6 (40)
	Nucleolar		1 (7)
SSc-specific antibodies	Scl-70		4 (27)
	RNA pol III		0
Vasculopathy, *N* (%) patients	Digital ulcers		6 (40)
	PAH		0
	Renal crisis		0
Interstitial lung disease, *N* (%)			4 (27)
Inflammatory arthritis, *N* (%)			3 (20)
Vascular therapies, *N* (%) patients	ARB		10 (66)
	Ca channel antagonist		4 (27)
	Iloprost		4 (27)
	PDE5 inhibitor		3 (20)
Immunosuppressive therapies, *N* (%) patients	PDN		4 (27)
	MMF		3 (20)
	MTX		2 (13)
	AZA		1 (7)
	HCQ		3 (20)

*ANA* antinuclear antibodies, *SSc* systemic sclerosis, *MRSS* modified Rodnan skin score, *PAH* pulmonary arterial hypertension, *ARB* angiotensin receptor blocker, *PDE* cGMP-regulated phosphodiesterase, *MTX* methotrexate, *HCQ* hydroxychloroquine, *PDN* prednisolone, *AZA* azathioprine

### Overview of gene expression profiles

**Fig. 1:**
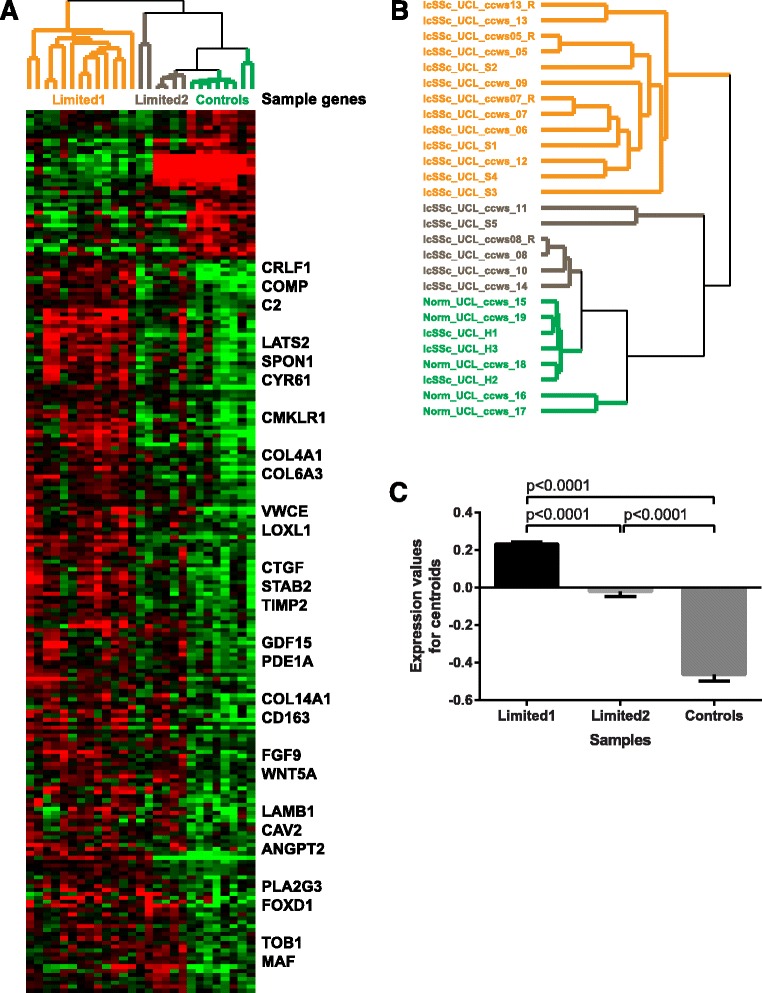
Gene expression in whole skin samples from patients with limited cutaneous systemic sclerosis (*lcSSc*) and healthy controls (*Controls*). **a** Heatmap: *red* indicates upregulation, *green* indicates downregulation. Gene expression in the *limited 1* subgroup of samples from patients with lcSSc differs markedly from both the *limited 2* subgroup and from healthy controls, which shared similar gene expression patterns. Sample genes of interest are listed. **b** Hierarchical clustering of genes distinguishing lcSSc samples from healthy control samples identifies three distinct clusters, termed limited 1, limited 2 and healthy control. *Norm* Normal. **c** Pairwise comparisons between limited 1, limited 2 and controls. Data are plotted as mean with SEM values. *P* values were derived from the Kruskal-Wallis test followed by Dunn's multiple comparisons test

There were 218 differentially expressed genes (DEG) between lcSSc and healthy control samples (false discovery rate (FDR) <10%). Of these 218 genes, 181 (83%) were upregulated in lcSSc samples and were significantly enriched in several terms related to the extracellular matrix (ECM), e.g. ECM organisation and ECM component and response to growth factor, tissue development and regulation of the serine/threonine kinase signalling pathway. This gene signature included genes previously implicated in the pathogenesis of SSc such as *ANGPT2*, *CD163*, *COMP*, *CTGF* and *TIMP2*, among others (Fig. 1a). The full list of significantly differentially expressed genes is available in Additional file 1.

Even though we specifically asked for genes differentially expressed between lcSSc samples and healthy controls, 6 lcSSc samples (5 unique patients) clustered with controls, whereas the remaining 13 lcSSc samples (10 unique patients) formed a distinct cluster (Fig. 1b). We designated 13 lcSSc samples as the “limited 1” group and 6 lcSSc samples as the “limited 2” group. The expression of 181 genes significantly upregulated in lcSSc vs. healthy controls was also significantly different in all pairwise comparisons between limited 1, limited 2 and control samples (Fig. 1c). Mean ± standard error of the mean expression values for 181 gene signatures were as follows: 0.23 ± 0.01 for limited 1, -0.02 ± 0.03 for limited 2 and -0.46 ± 0.03 for healthy control samples.

**Fig. 2:**
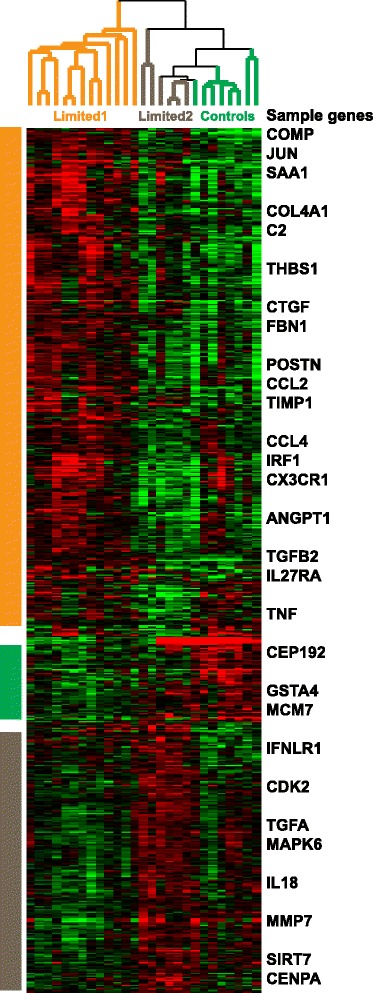
Multiclass significance analysis of microarrays (SAM) between the three identified clusters. Multiclass SAM was used to identify 807 differentially expressed genes (false discovery rate <10%) between limited -1, limited -2 and healthy control samples. Sample genes are listed. The majority of differentially expressed genes (475/807, 58.9%) had increased expression in the limited-1 group

Since comparison between lcSSc and controls was suggestive of the presence of two lcSSc groups, we performed multiclass SAM to identify DEG between limited-1, limited-2 and healthy control samples: 807 genes were differentially expressed between these three groups (FDR <10%). Again, limited-2 samples clustered with controls and separately from the limited-1 group (Fig. 2; see Additional file 2 for the full gene list).

Genes with significantly increased expression in either the limited-2 group or in healthy controls were not functionally enriched. Genes with significantly increased expression in samples from the limited-1 group (475/807, 58.9%) displayed very strong enrichment in functional terms related to ECM and vasculature development (*p* < 10^-10^) and strong enrichment in cell adhesion, response to growth factor, mitogen-activated protein kinase (MAPK) cascade and response to wounding (*p* < 10^-5^), among others. Enrichment in immune signalling was comparatively weak (e.g. *p* = 0.0183 for immune system process and *p* = 0.0208 for inflammatory response). The entire g:Profiler output is listed in Additional file 3.

For the limited-1 group, the term with the most significant functional enrichment was cardiovascular system development (*p* = 3.01 × 10^-14^): 70 out of 475 genes with increased expression in limited-1 samples were involved in this biological process. This set of 70 genes essentially recaptured many of the biological themes observed in the bigger limited-1 gene expression signature. For example, there was very strong enrichment in angiogenesis, response to growth factor and cell proliferation (*p* < 10^-10^) and strong enrichment in ECM, cartilage development, MAPK cascade, cell adhesion and response to wounding (*p* < 10^-5^), among others. It was also strongly enriched in response to transforming growth factor (TGF)-β (*p* = 2.87 × 10^-7^, 12/70 genes). Additional file 4 contains the entire g:Profiler output for this analysis. There were no significant differences in demographic, clinical or serological parameters that correlated with limited-1 and limited-2 groups, in particular, the presence of significant microvascular involvement and hallmark antibody reactivities were similar across each group as shown in Table 2 below.

**Table 2:** Clinical and laboratory parameters of the limited-1 and limited-2 subgroups

Clinical feature		Limited 1 (*n* = 10)	Limited 2 (*n* = 5)
MRSS, median (range)		5.5 (3–10)	6 (5 − 7)
Disease duration from first non-Raynaud’s symptom, median (range) years		16 (4–40)	6 (2–28)
ANA primary pattern, *N* (%) patients	Homogenous	3 (30)	1 (20)
	Speckled	1 (10)	1 (20)
	Centromere	5 (50)	1 (20)
	Nucleolar	0	1 (20)
SSc-specific antibodies	Scl-70	3 (30)	1 (10)
	RNA pol III	0	0
Vasculopathy, *N* (%) patients	Digital ulcers	4 (40)	2 (40)
	PAH	0	0
	Renal crisis	0	0
Interstitial lung disease, *N* (%)		2 (20)	2 (40)
Inflammatory arthritis, *N* (%)		3 (30)	0
Vascular therapies, *N* (%) patients	ARB	8 (80)	2 (40)
	Ca channel antagonist	2 (20)	2 (40)
	Iloprost	3 (30)	1 (20)
	PDE5 inhibitor	1 (10)	2 (40)
Immunosuppressive therapies, *N* (%) patients	PDN	2 (20)	2 (40)
	MMF	2 (20)	1 (20)
	MTX	2 (20)	0
	AZA	1 (10)	0
	HCQ	2 (20)	1 (20)

*ANA* antinuclear antibodies, *SSc* systemic sclerosis, *MRSS* modified Rodnan skin score, *PAH* pulmonary arterial hypertension, *ARB* angiotensin receptor blocker, *PDE* cGMP-regulated phosphodiesterase, *MTX* methotrexate, *HCQ* hydroxychloroquine, *PDN* prednisolone, *AZA* azathioprine

### Validation of microarray gene expression profiles in an independent lcSSc cohort

**Fig. 3:**
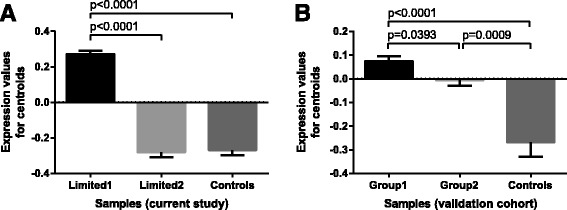
Cardiovascular development trends in discovery and validation cohorts. Pairwise comparisons between limited 1, limited 2 and control samples in the discovery cohort (**a**) and an independent validation cohort (**b**). Data are plotted as mean with SEM values. *P* values were derived from the Kruskal-Wallis test followed by Dunn's multiple comparisons test. *CVS* cardiovascular system

We applied the same approach described above to validate our findings in an independent cohort of patients with lcSSc derived from an American population (the Boston University cohort), with samples comprising 24 lcSSc and 4 control samples. Similar to this study cohort, analysis of differential expression between lcSSc and control samples suggested the presence of two subgroups of patients with lcSSc (group 1 with 14 lcSSc samples and group 2 with 10 lcSSc samples). We then compared the gene expression of the CVS development gene signature across subgroups of lcSSc patients and controls from the validation study. This gene expression signature was significantly different among both subgroups of patients with LcSSc and a control group (Fig. 3b), paralleling the results from this study (Fig. 3a).

## Discussion

In this study we have assessed molecular heterogeneity in gene expression profiles from skin biopsies taken from uninvolved skin of lcSSc patients compared with matched healthy controls. We have identified two subgroups within the lcSSc patient population in this study, which we termed limited 1 and limited 2. Patients from the limited-2 subgroup correspond to a third of the lcSSc cases and are generally characterised by a subtle alteration in gene expression that resembles but is distinct from the control samples. The majority of lcSSc cases cluster into a limited-1 subgroup associated with a substantial number of genes with significant differential expression, which are involved in multiple functional terms with known or potential relevance to SSc pathogenesis. We have confirmed our findings in a second cohort.

Most compelling is the identification of a cardiovascular system (CVS) development gene expression signature. This is interesting because of the well-recognised vascular abnormalities that are seen in lcSSc [3]. The idea that these pathways may be upregulated or altered in lcSSc even at sites that are not clinically affected is notable and may point towards an inherent susceptibility to vasculopathy that may be a hallmark of SSc. It is plausible that using uninvolved lcSSc skin we were able to minimise the impact of pathways reflecting inflammation and that this may explain the novel findings in our study. It was reassuring to find that several genes identified within this group were well-recognised in scleroderma pathogenesis, including, for instance, *COMP*, *THBS1* and *CTGF*. These have traditionally been considered as pro-fibrotic markers, but all have a role in the regulation of vascular function [23–25]. More traditional “vascular” markers such as *MMP19* and *COL4A1* were also included. It is also attractive to consider that recapitulation of developmental gene expression programmes may be central to susceptibility to development of SSc since it can be envisaged as a disease of perturbed connective tissue repair. There is a strong precedent for the same genetic pathways and programmes that are used in embryonic and post-natal growth and development to be recapitulated in acquired disease [26]. This may also fit with the model of SSc being a susceptibility genotype that we have recently postulated for the key complication of pulmonary arterial hypertension [27].

It is also notable that there were no clear clinical or serological associations with the molecular subgroups of lcSSc. This is relevant since it suggests that gene-expression-based subsets may indeed add to the clinical and serological factors that are already used in the clinic to subgroup patients. This would be analogous to the intrinsic subsets of diffuse cutaneous SSc (dcSSc) that have been reported and that are already of clinical utility [7]. Of note, in the cohort from which the study samples were taken, 40% of patients with limited disease were anti-topoisomerase (Scl-70)-positive and while this is traditionally thought to represent diffuse scleroderma, the patients in this cohort are categorised according to the distribution of skin disease at an early stage of disease and retain that subset over the course of their follow-up period. There were, therefore, no patients included in this study who would have experienced remission to a low skin score with previous diffuse or more severe skin disease.

It is striking that many of the genes involved in CVS development are also seen in other relevant pathways such as ECM and response to TGF-β. Not only does this give some key candidate genes and pathways that could be perturbed in SSc but it also is a reminder that these factors that have been focussed on for a role in fibrosis may also have other roles in the biology or the disease. Thus it is plausible that the patterns of gene expression are altered in SSc and that this may have distinct consequences depending upon the stage and subset of disease. Further work will be required to assess whether these differences in expression reflect susceptibility to SSc that depends on genetic or epigenetic factors or whether they are reflective of pathogenetic processes that are occurring within the clinically uninvolved skin in limited SSc.

Strengths of the study include the well-characterised patients from a single centre, which may reduce the variability that can be a hallmark of multicentre cohorts and ensures standard classification and clinical evaluation. In addition, the use of well-established gene expression analysis platforms and semi-automated sample processing minimises technical variation that has been a limitation in some studies. Inclusion of a matched control group of healthy individuals was important to allow reliable interpretation of the “limited” gene expression signature.

Weaknesses of the study are the limitations of a relatively small study cohort that may not allow generalisability of our findings. However, the results are in line with those previously reported for subjects with lcSSc included in earlier gene expression studies [7]. We have validated our results in an independent cohort of patients with consistent findings across all groups. There is also a risk, given the small size of the study that there may be confounding effects on gene expression due to differences in immunosuppressive therapies, as an example. There were no statistically significant differences or strong trends between the limited-1 and limited-2 groups in terms of demographics, clinical history, treatments or serology in our study.

We have not included patients with clinically involved forearm skin in this study. There are no adequately powered studies that confirm concordance between clinically involved and uninvolved skin but previous data do suggest this; these confirmatory studies are required to extend and validate our findings further as part of a larger future study of gene expression in lcSSc. Involved distal skin is not biopsied in our patients due to concerns about wound healing. Finally, both further post-transcriptomic functional studies, and modern techniques that allow structural analysis of mRNA expression most notably to examine the dermal microcirculation and perivascular space would localise gene expression changes and verify that differential gene expression, particularly from the CVS development cluster, are reflected by differential protein expression.

## Conclusions

We showed that gene expression profiling of biopsies from uninvolved skin in lcSSc differentiates two potential subgroups that overlap with other clinical and serological features. This may reflect important differences in pathogenesis within these patient groups. In addition, we identified differential expression of a subset of genes that relate to CVS development. Since the lcSSc subset is characterised by vasculopathy in the skin and internal organs, this may shed light on underlying mechanisms of vascular disease in SSc. The clinical implications of our findings will need to be analysed in future larger studies.

## Additional files

Additional file 1: Genes significantly differentially expressed between lcSSc and control groups. Green cells: genes with increased expression in controls. Yellow cells: genes with increased expression in lcSSc samples. (XLSX 16 kb)

Additional file 2: Genes significantly differentially expressed between the limited-1, limited-2 and control groups. Orange cells: genes with increased expression in limited 1 group. Green cells: genes with increased expression in controls. Grey cells: genes with increased expression in limited 2 group. (XLSX 38 kb)

Additional file 3: g:Profiler output for 475 genes with increased expression in the limited-1 group (multiclass SAM, FDR <10%). Additional files 3 contain the following column headers. Descriptions are as follows: *p* value: significance of enrichment in a given term corrected for multiple hypothesis testing using default g:GOSt method g:SCS. Q&T: overlap between genes in the query (Q) and genes in the genome annotated to a given term (T). t type: term category - Gene Ontology: Biological Process (BP), Molecular Function (MF), Cellular Component (CC); KEGG (keg); Reactome (rea). t name: term name. Q&T list: list of genes forming the overlap between query (Q) and genome lists for a given term (T). (XLSX 25 kb)

Additional file 4: g:Profiler output for 70 genes with increased expression in the limited-1 group annotated to cardiovascular system development. Additional file 4 contains the following column headers. Descriptions are as follows: *p* value: significance of enrichment in a given term corrected for multiple hypothesis testing using default g:GOSt method g:SCS. Q&T: overlap between genes in the query (Q) and genes in the genome annotated to a given term (T). t type: term category - Gene Ontology: Biological Process (BP), Molecular Function (MF), Cellular Component (CC); KEGG (keg); Reactome (rea). t name: term name. Q&T list: list of genes forming the overlap between query (Q) and genome lists for a given term (T). (XLSX 29 kb)

## Abbreviations

ANA: Antinuclear antibody
AZA: azathioprine
CVS: Cardiovascular system
dcSSc: Diffuse cutaneous systemic sclerosis
DEG: Differentially expressed genes
ECM: Extracellular matrix
ENA: extractable nuclear antigen
EULAR: European League Against Rheumatism
FDR: False discovery rate
HC: Healthy controls
IIF: Indirect immunofluorescence
lcSSc: Limited cutaneous systemic sclerosis
MAPK: Mitogen-activated protein kinase
MRSS: Modified Rodnan skin score
PDN: prednisolone
SAM: Significance analysis of microarrays
SSc: Systemic sclerosis
TGF: Transforming growth factor
